# Smart hospital: achieving interoperability and raw data collection from medical devices in clinical routine

**DOI:** 10.3389/fdgth.2024.1341475

**Published:** 2024-03-06

**Authors:** Eimo Martens, Hans-Ulrich Haase, Giulio Mastella, Andreas Henkel, Christoph Spinner, Franziska Hahn, Congyu Zou, Augusto Fava Sanches, Julia Allescher, Daniel Heid, Elena Strauss, Melanie-Maria Maier, Mark Lachmann, Georg Schmidt, Dominik Westphal, Tobias Haufe, David Federle, Daniel Rueckert, Martin Boeker, Matthias Becker, Karl-Ludwig Laugwitz, Alexander Steger, Alexander Müller

**Affiliations:** ^1^ TUM School of Medicine and Health, Department of Clinical Medicine—Clinical Department for Internal Medicine I, University Medical Center, Technical University of Munich, Munich, Germany; ^2^ European Reference Network Guard Heart, European Union, Amsterdam, Netherlands; ^3^ TUM School of Medicine and Health, Department of Clinical Medicine—Department of Information Technology, University Medical Center, Technical University of Munich, Munich, Germany; ^4^ IHE Deutschland e.V, Berlin, Germany; ^5^ TUM School of Medicine and Health, Department of Clinical Medicine—Clinical Department for Internal Medicine II, University Medical Center, Technical University of Munich, Munich, Germany; ^6^ Working Group of Medical Ethics Committees in the Federal Republic of Germany e.V., Berlin, Germany; ^7^ TUM School of Medicine and Health, Department of Clinical Medicine—Ethics Committee, University Medical Center, Technical University of Munich, Munich, Germany; ^8^ TUM School of Medicine and Health, Department of Clinical Medicine—Clinical Department for Human Genetics, University Medical Center, Technical University of Munich, Munich, Germany; ^9^ TUM School of Medicine and Health, Center for Digital Health & Technology—Institute for Artificial Intelligence and Informatics in Medicine, University Medical Center, Technical University of Munich, Munich, Germany; ^10^ Department of Computing, Imperial College London, London, United Kingdom; ^11^ Development Department, Fleischhacker GmbH & Co, Schwerte, Germany; ^12^ German Center of Cardio-Vascular-Research (DZHK), Berlin, Germany

**Keywords:** interoperability, clinical information system, medical device data, FHIR, biosignals, data integration

## Abstract

**Introduction:**

Today, modern technology is used to diagnose and treat cardiovascular disease. These medical devices provide exact measures and raw data such as imaging data or biosignals. So far, the Broad Integration of These Health Data into Hospital Information Technology Structures—Especially in Germany—is Lacking, and if data integration takes place, only non-Evaluable Findings are Usually Integrated into the Hospital Information Technology Structures. A Comprehensive Integration of raw Data and Structured Medical Information has not yet Been Established. The aim of this project was to design and implement an interoperable database (cardio-vascular-information-system, CVIS) for the automated integration of al medical device data (parameters and raw data) in cardio-vascular medicine.

**Methods:**

The CVIS serves as a data integration and preparation system at the interface between the various devices and the hospital IT infrastructure. In our project, we were able to establish a database with integration of proprietary device interfaces, which could be integrated into the electronic health record (EHR) with various HL7 and web interfaces.

**Results:**

In the period between 1.7.2020 and 30.6.2022, the data integrated into this database were evaluated. During this time, 114,858 patients were automatically included in the database and medical data of 50,295 of them were entered. For technical examinations, more than 4.5 million readings (an average of 28.5 per examination) and 684,696 image data and raw signals (28,935 ECG files, 655,761 structured reports, 91,113 x-ray objects, 559,648 ultrasound objects in 54 different examination types, 5,000 endoscopy objects) were integrated into the database. Over 10.2 million bidirectional HL7 messages (approximately 14,000/day) were successfully processed. 98,458 documents were transferred to the central document management system, 55,154 materials (average 7.77 per order) were recorded and stored in the database, 21,196 diagnoses and 50,353 services/OPS were recorded and transferred. On average, 3.3 examinations per patient were recorded; in addition, there are an average of 13 laboratory examinations.

**Discussion:**

Fully automated data integration from medical devices including the raw data is feasible and already creates a comprehensive database for multimodal modern analysis approaches in a short time. This is the basis for national and international projects by extracting research data using FHIR.

## Introduction

Medical research and care is based on clinical experience and the results of anamnesis, physical and technical examinations. Technical examinations are carried out with medical devices, which have become increasingly sophisticated in recent years (1). In most cases, a large number of measurements, settings and raw image or biosignal data are recorded. In addition, reporting is performed with different software solutions. This forms the basis for further clinical decisions. So far, this evaluation has been done—if at all—in partially structured settings with a high proportion of unstructured information (2). Unfortunately, poorly structured data are not suitable for modern data analysis using automated computing technologies such as machine learning algorithms to support diagnostic and therapeutic decision making (3). From a scientific perspective, digitally acquired measurements are still often documented by hand and transferred again by hand from this documentation into special, digital study systems for research (4). In addition to the poor data transmission, another major problem can be identified: there is no uniform designation for the measured values for data exchange (semantic interoperability), as the vast majority of manufacturers only use proprietary designations for the parameters, but there are also no interoperability standards for many of these measured values (e.g., SNOMED-CT or LOINC codes) (5, 6). Thus, despite a very high standard of technology in medical technology, there is a relevant gap in the automation of data collection and structured storage, which represents a considerable impairment of treatment quality and a clear hurdle for modern IT procedures in health care and research (7, 8). In addition to medical devices and paper documentation, many hospitals have special non-interoperable subsystems in which documentation takes place (9). Systems established so far are usually established for special clinical and research questions or special devices; a comprehensive system for medical device integration and structured data collection even only for the field of cardiology has not been established (10, 11).

### Aim of the project

**Figure 1:**
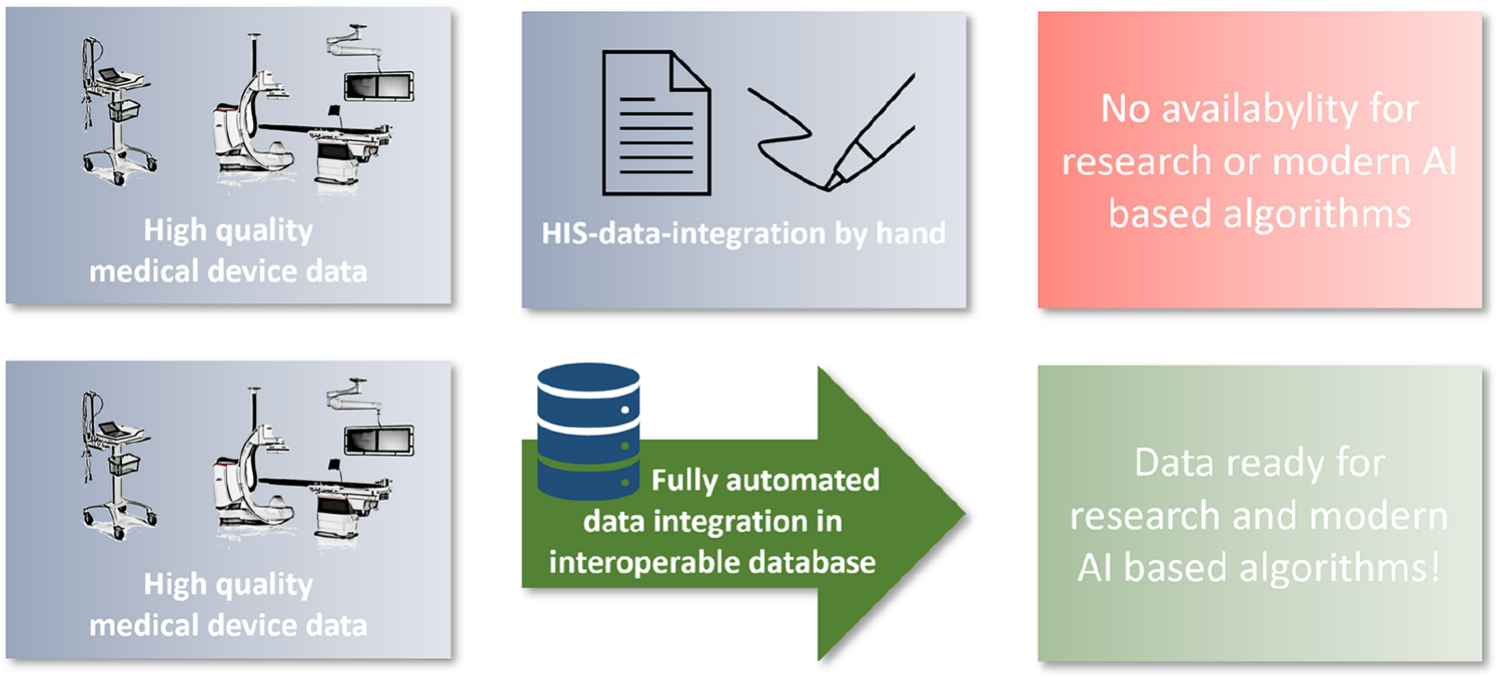
Central illustration.

The aim of this project was to establish a cardio-vascular information system (CVIS) with integration of as many medical devices as possible and structured reporting based on clinical and scientific requirements. This system should be integrated as deeply as possible into the clinical IT landscape and be able to make the data available again for different purposes (research and patient care) in an interoperable way. The EHR serves as a data integration and preparation system at the interface between the various devices and the hospital IT infrastructure. Special focus was placed on the subsequent provision of structured data for AI models (Figure 1).

## Material and methods

### Requirements analysis

At the beginning of the project, a needs analysis was carried out on the basis of the existing systems in the pilot department (cardiology) and the clinical and scientific requirements, with a detailed list of the existing medical devices, special software systems and clinical documentation processes. The analysis was based on two different areas. In the clinical part, a structured interview was conducted with the doctors and nursing staff involved in the respective department, taking into account the previous clinical workflows. This interview focused on querying existing clinical processes and the technical conditions on site. In addition, the optimal workflow from a clinical perspective was also inquired about. In the second step, a systematic literature search (based on a standardised meta-analysis, PubeMed, Google Scholar, IEEE) was conducted for the respective department (e.g., clinical documentation electrophysiology, cardiology, cardiac catheterization, echocardiography, angiology, pneumology, etc.). The search results were processed according to methods and clinical implementation and agreed in working groups. Finally, the results of the two areas were summarised in a specialist concept for each functional area and finalised together. Additionally, the technical connection to the existing, central clinical information systems and thus to the GEMATIK (Society for Telematics Applications of the Health Card in Germany) infrastructure was analysed on the same way. As an important research component, the interoperable data transfer to existing research systems but also to national and international databases (e.g., Medical Informatics Initiative, European Reference Network) was assessed.

### Implementation

The conduction of the project was divided into five different fields: IT environment, hospital EMR structures integration, structured reporting, medical device integration and research data derivation.

### IT environment

After planning the future application environment, a total of 8 virtual machines were set up in a central virtual machine (VM) ware environment. In addition, storage was made available in the central NAS infrastructure with corresponding backup (55TB). In addition, the capacities of the central image archive (PACS) were expanded as long-term storage. All databases are in an SQL database environment (MS SQL 2016).

### Integration into the hospital it landscape

**Figure 2:**
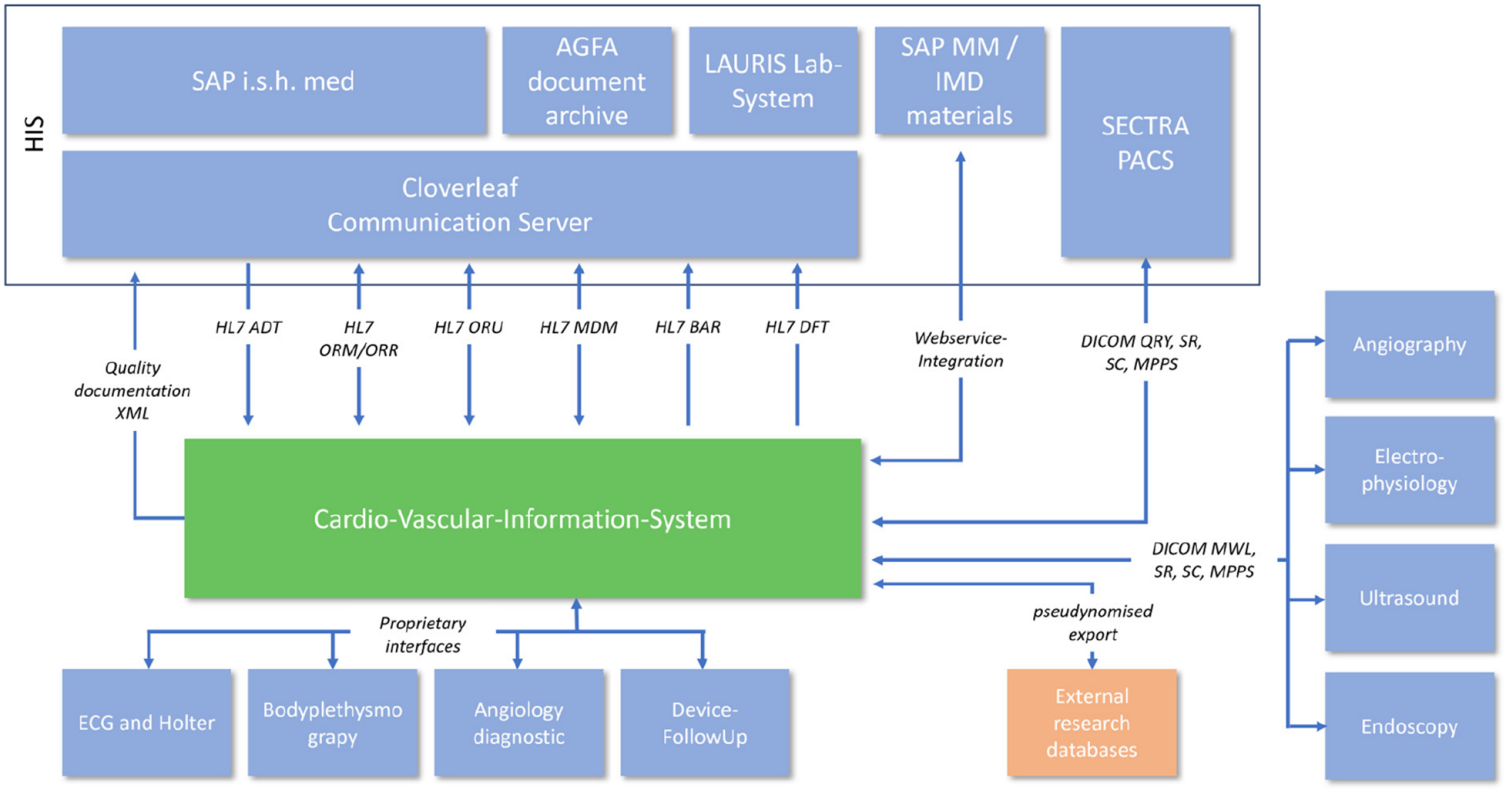
System interface overview.

The integration into the hospital EMR landscape has many different facets. In addition to the possibility of automatic transfer of diagnoses and services, a large number of different interfaces were necessary depending on the area of application (Figure 2).

### HL7 interfaces

Analysis of the existing hospital infrastructure revealed that a large part of the integration had to be realized via internationally standardized Healthcare Level 7 (HL7) Version 2.x interfaces. The administrative processing of patient data remained in the main clinical EMR system SAP R3/Integrated Solution Healthcare (IS-H) in combination with Oracle-Cerner i.s.h.med, Idstein, Germany). The communication path begins with the creation of a clinical order in SAP IS-H, which had to be redesigned for this project. A summary of the HL7 interfaces is shown in Table 1.

**Table 1:** HL7 interfaces.

HL7 interface	Description	Comm.-direction
		HIS >CVIS
ORM	Order message	CVIS >PACS
ADT	Admission, discharge, transfer	HIS >CVIS
ORR	Order response, Statusnachrichten	CVIS >HIS
ORU	Observation result unsolicited	CVIS <>HIS
		CVIS >PACS
MDM	Medical document management	CVIS <>HIS
DFT	Detailed financial transactions	CVIS >HIS
BAR	Billing account record	CVIS >HIS

### Digital imaging and communications in medicine (DICOM)

All image data from medical devices that are integrated into the cardio-vascular information-system (CVIS) are directly sent to (after initial HL7 V2.x Order Message, ORM) and stored in the EMR's Picture Archiving and Communication System (PACS, SectraAB, Sweden). The image data can then be retrieved from the archive using DICOM Query/Retrive.

### Integration of material acquisition

Consumable material also plays an important role in structured documentation. The material data helps with the further automation of process sequences as well as exact documentation and traceability. We have integrated the existing SAP R3/MM based material documentation system into the new process and system via web service. All consumables are scanned, stored in SAP R3/MM data base and transferred to CVIS via web service for further documentation and processing.

### Quality assurance

In Germany, quality assurance is required by national law for medical procedures [§ Section 135a (1) SGB V] and standardized for various invasive procedures such as many cardiovascular interventions and operations. Most of the required information is already collected during the treatment or examination process and can be further used for the quality assurance documentation. For the export of this documentation, an XML export was created in the quality assurance system (KAP GmbH, SAP module, Berlin, Germany), which automatically transmits the corresponding data to the Institute for Quality Assurance and Transparency in Health Care.

### Structured reporting

Based on the needs analysis, so-called workflows for structured reporting were created for all examination types and use cases as well as for different anamnesis scenarios. Table 2 provides an overview of the different workflows. During planning, the requirements of the individual functional departments were compared with each other in order to realize the highest possible degree of standardization. In addition to medical documentation, nursing documentation also takes place in the new workflows (Table 3). Input masks for the same parameters (e.g., vital signs documentation) can thus always be operated in the same way and contain the same structured parameters. The existing semantic interoperability standards were taken into account during the creation as well as the subsequent export to existing (external) research registers.

**Table 2:** Clinical workflows.

Discipline	Workflows for structured data collection
Angiology	Angiological examination/ultrasound
	Peripheral catheter intervention
Cardiology	Cardiac catheterisation
	Hospital admission
	Interventional valve therapy
Electrophysiology	Cardioversion
	Device Implantation
	Device-FollowUP InClinic
	Electrophysiological examination and ablation
	Cardio-genetics
Pneumology	Bronchoscopy
	Pleural sonography
Telemedicine	Quality of life questionnaire
	Study inclusion workflow
	Study screening log
	Study visit workflow
	Tele-visit
	Device-FollowUP remote

**Table 3:** Examination-types.

Technical investigation	Raw data type
Abbott Merlin.net Importer	Biosignals, device-data
Abdominal sonography	Images, structured report
ABI Messurement	Structured data
Angiography (x-Ray)	Images, structured report
Biotronik HomeMonitoring Importer	Biosignals, device-data
Boston Latitude Importer	Biosignals, device-data
Bronchoscopy (Endoscopy & x-Ray)	Images, structured report
Capillary microscopy	Images, structured report
CO2 Test	Structured data
Device FollowUP Abbott	Biosignals, device-data
Device FollowUP Biotronik	Biosignals, device-data
Device FollowUP Boston Scientific	Biosignals, device-data
Device FollowUP Medtronic	Biosignals, device-data
Device FollowUP Microport	Biosignals, device-data
DICOM Query	Images
Doppler ultrasound of the brain-supplying vessels	Images, structured report
Doppler ultrasound of the cerebral vessels	Images, structured report
Doppler ultrasound of the extremities	Images, structured report
Electrophysiology Measurement System	Biosignals, structured data
Fractional Flow Reserve (FFR)	Structured data
Holter-EKG	Biosignals, structured data
Holter-RR	Strucutred data
Intravascular Ultrasound (IVUS)	Images, structured report
Light reflection rheography (LRR)	Images, structured report
Medtronic CareLink Importer	Biosignals, device-data
Optical coherence tomography	Images, structured report
Optical Pulse Oscillography	Images, structured report
Oscillography	Images, structured report
Resting ECG	Biosignals, structured data
Rhythm ECG	Biosignals, structured data
Schwarzer EVO– Haemodynamics	Biosignals, structured data
Siemens Sensis—Haemodynamics	Biosignals, structured data
Stress Echocardiography	Images, structured report
Stress ergometry	Biosignals, structured data
TcPO2 Measurement	Structured data
Transoesophageal Echocardiography (TEE)	Images, structured report
Transthoracal Echocardiography (TTE)	Images, structured report
Venous function diagnostics	Images, structured data
Wound documentation	Images

### Medical device integration

The largest part of the project involved the integration of medical devices including measurements, biosignal data and image data. For this purpose, the existing infrastructure was analyzed and, in a first step, it was determined which existing devices were capable of exporting raw data. Subsequently, partially new interfaces and new devices were procured if digital data export was not possible. In the next step, the interfaces of all devices (cf. Table 3) were analyzed. For some devices, already available “standardized” interfaces such as DICOM or HL7 2.X could be used. However, in the vast majority of devices, connection via proprietary interfaces was necessary. The aim was to import measured values and parameters as well as raw data from biosignals or image data as available. Within the framework of the project, all previously used telemedical systems were also connected and integrated.

### Research data export

Throughout the project, the use of the collected data in the research context has a very high priority. For this purpose, the possibility of manual and automatic data export was created. Manually, all users with the appropriate authorisation can select and filter all fields visible in the system from the SQL database via a drag-and-drop module and thus export them for research projects. Here, the fast and also combined evaluation of data from different data sources is particularly important. For the acquisition of study patients, automated study dashboards were created to display potential study patients based on inclusion and exclusion criteria.

In addition to the analysis directly on the database, the automatic transfer of data to research registers or research databases plays an essential role in simplifying scientific processes. Registries of the European Reference Network (XML export), the German Centre for Cardiovascular Research (XML export) and the ISAR Research Centre (direct database export) have already been connected.

### Interoperability

All data from the different devices are normalised during import and thus stored in an interoperable way. If interoperability standards are already available for the parameters and structured values, the recorded parameters are mapped according to SNOMED-CT (Systematised Nomenclature of Medicine) and/or LOINC (Logical Observation Identifiers Names and Codes) and stored in a structured way. All clinical parameters recorded in a structured way are also stored in a semantically interoperable way. LOINC, SNOMED-CT or FHIR profiles are therefore the “languages” into which a translation is already available in the database. The system is adapted to new versions of LOINC, SNOMED-CT or FHIR profiles several times a year. Overall, however, corresponding semantic interoperability codes exist so far only for a very small part of the recorded parameters. All other parameters are stored in normalised form and can later be provided with corresponding codes as soon as coding systems or profiles are available.

The period from 1.7.2020 to 30.6.2022 was used for the evaluation.

## Results

### Project implementation

The application and initial requirements planning were completed between May 2017 and January 2018. After positive funding approval (3/2018) from the German Research Foundation (DFG), a market survey was initially carried out and then a Europe-wide call for tenders was prepared and carried out in a bidding competition. The contract was awarded in March2019.

The implementation of the entire connection, from the first installation of a virtual machine to the last medical device connection and corresponding staff training, took from 04/2019 to 03/2020. The implementation was carried out according to a defined multi-stage plan.

### Implementation results

A total of 18 diagnostic workflows were created and put into operation (cf. Table 2) and seven HL7 interfaces with the leading hospital information system were designed, programmed and put into operation (cf. Table 1). In the project, we were able to establish comprehensive HL7 integration for master data communication (ADT), order communication (ORM/ORR), transmission of findings for further use (ORU), document transmission (MDM), diagnosis communication (BAR) and service communication (DFT). In addition to communication with the HIS (SAP i.s.h. med via communication server), order (ORM) and findings communication (ORU) with the PACS (Sectra) was established to ensure consistent availability of the findings.

In addition, the DICOM connection to the PACS and a web service for communication with materials management were established. For data export, a HL7 FHIR interface and the possibility of CDA PDF export were created. In addition, older/proprietary registers were connected with an XML interface (Figure 2). The implementation of the HL7 infrastructure took up a considerable part of the project period (approx. 1 year) and corresponding financial resources.

The largest part of the project was the connection of different devices. A total of >115 different medical devices were connected in 39 modalities. A special part of the project was the connection of the mostly proprietary interfaces and the normalization of the parameters. Even with supposedly standardized communication interfaces such as DICOM, adjustments were often necessary, as the structured reports are not standardized and the image data can also be transferred with different parameters. All of these interfaces therefore had to be processed individually in order to ensure a standardized data basis.

### Operating results

In the observed 2 years after the introduction of the system, data for a total of 114,858 patients were recorded in the new Cardiovascular Information System (CVIS). For a total of 50,295 patients, cardiovascular examinations were also performed—for the remaining 64,563, only laboratory data were recorded. One hundred sixty-nine thousand four hundred fifty-nine orders with technical examinations and diagnostic workflows were successfully created, processed and released. One million six hundred three thousand one hundred ninety-eight laboratory examinations were submitted. In the technical examinations, more than 4.5 Mio measured values (on average 28.5 per examination) and 684,696 image data and raw signals (28,935 ECG files, 655,761 structured reports, 91,113 x-ray objects, 559,648 ultrasound objects in 54 different examination types, 5,000 endoscopy objects) were integrated into the database (Table 4). Over 10.2 million HL7 messages (approx. 14,000/day) were successfully processed inbound or outbound. Ninety-eight thousand four hundred fifty-eight documents were transferred to the central document management system, 55.154 materials (average 7.77 per order) were recorded and stored in the database, 21,196 diagnoses and 50,353 services/OPS were recorded and transferred.

**Table 4:** Top examinations.

	Standardized variables	Measured parameters	Procedures	Measurements/procedure
Resting ECG	43	1.919.485	84.433	22.73
Pacemaker/ICD	707	1.690.737	23.060	73.32
Echocardiography	459	371.762	21.276	17.47
Haemodynamics	249	163.731	8.608	19.02
Angiology diagnostic	438	137.603	7.296	18.86
Long term ECG/RR	145	112.811	6.149	18.35
Angiography	23	28.971	3.937	7.36
Ergometry	52	4.490	498	9.02

On average, 3.3 examinations are recorded per patient; plus an average of 13 laboratory examinations.

## Digital transformation

In addition to the technical implementation, the digital transformation of the employees was also a key aspect of the project, which had a major impact on the project. This affected two areas in particular: the switch from paper-based documentation to digital documentation and the switch from free-text documentation to structured recording of findings.

## First use-cases

During the first 2 years of project implementation, we were able to design and finalize various scientific papers based on the interoperable database. In some cases, special study workflows or electronic case report forms (eCRFs) were also created, filled and analyzed in the structured database (13–16).

In these initial use cases, we were able to demonstrate the advantages of standardized data integration, e.g., of measured values from the cardiac catheter. In addition, structured reporting for automated data retrieval proved to be a fundamental advantage in terms of data comparability and the ability to make data available at short notice.

In the use cases with data from different modalities, these were also collected with different types of devices (e.g., ultrasound, lung function or echocardiography) from different manufacturers. This data was made available for the use cases in the database using LOINC coding, regardless of the manufacturer. In these initial use cases, we have already been able to demonstrate the added value of the interoperable database and thus contribute to rapid scientific evaluation.

## Discussion

Cardiovascular disease and sudden cardiac death are still extremely common causes of death in the western world (17). The treatment of this spectrum of diseases is strongly structured by guidelines and evidence-based scoring systems (18). In addition, intersectoral network structures for the care of patients with these diseases are recommended by the guidelines and certified by the professional societies (19).

So far, there has been a technical gap between the medical requirements for networking and decision support and reality. Currently, the parameters for the scores have to be entered manually into mostly web- or app-based calculators (20). Likewise, the intersectoral exchange between the various service providers in patient care still takes place by means of paper printouts and faxes (21). Many countries such as Sweden have been working with intersectoral electronic patient records for years, but the automated exchange of structured data (e.g., via CDA document or HL7 FHIR) has not been established yet (22).

Medical devices perform measurements and record biosignals or image data. So far, these modern devices have hardly been integrated into hospital IT systems or research systems with interoperable standards, so that they have to be transmitted manually again and again and the data are not available for modern algorithms and decision support systems. In addition, these data are often not available in the in-hospital treatment process or in intersectoral care (21).

In our project, we were able to show that it is possible to automatically integrate modern medical devices in everyday clinical practice into a structured and interoperable database and to record the findings in a structured way. In addition, we were also able to connect modern telemedical procedures including their biosignals. A special feature of the installation in our project is the comprehensive integration of all medical devices in cardiology as well as the comprehensive integration into the clinical IT system in order to cause as few media breaks as possible in the treatment process and to make the data available in intersectoral exchange via the GEMATIK infrastructure.

Regarding research, it is now possible to automatically transfer clinically collected routine data to and from big research databases. This does not require any new data entry into electronic study protocols. This also significantly improves quality and efficiency in the research context. In particular, the previously missing normalisation and structuring of data was a hurdle for such exports. In the project, it was important to consider the research questions already in the planning of the so-called workflows and also to have the possibility to adapt them further in the course of the project in order to be able to incorporate future questions. The.net framework platform on which the system is built leaves a lot of scope for future questions.

For future projects, there is the possibility of exporting via HL7 Fast Healthcare Interoberability Ressources (FHIR), in which case the new database functions as an FHIR repository in different Use Cases like Medical Informatics Initiative in Germany or other big data projects. The FHIR interface standard supports data exchange between different software systems in the healthcare sector, combining advantages from previous hospital communication (HL7 2.x) with modern web interfaces that have already been comprehensively implemented in other areas, thereby improving implementability. This creates the basis for sustainable data exchange and corresponding scalability (12).

Compared to other installations or systems, the requirement today is interoperable networking, but so far there are only a few systems on the market that include such extensive device integration and can also be used as a medical product for patient care. In addition, there is no such comprehensive medical device integration in a clinical information system that would be comparable with this project.

## Conclusion

In summary, we can state that with a funded project we were able to implement an interoperable database for structured reporting and integration of all device data. The system is able to integrate and forward data for clinical and scientific purposes and represents a comprehensive platform that simplifies many IT processes in patient care. There are of course still certain limitations, for example the project has so far only been limited to cardiovascular data integration and a lot of work still needs to be invested in the development of standardized data models, particularly in the area of interoperable data transfer, e.g., with HL7 FHIR. Following the project, we are rolling out the system on an interdisciplinary basis and we are part of the national standardization initiative for FHIR.

## Data Availability

The raw data supporting the conclusions of this article will be made available by the authors, without undue reservation.
